# Temperature should not be ignored when analyzing the factors affecting trauma outcomes

**DOI:** 10.1186/s13049-025-01363-w

**Published:** 2025-03-19

**Authors:** Yuliu Li, Tian Gu, Hongbing Yin

**Affiliations:** 1 Changchun University of Chinese Medicine, Changchun, China; 2 The Third Affiliated Hospital of Changchun University of Chinese Medicine, Changchun, China

Dear Editor,

We read with interest the analysis of Bin Kunji Mohamad MI et al. of gender-based influences on trauma outcomes among multiple trauma patients in the Asia-Pacific region [1]. The authors employed a hierarchical analysis utilizing a substantial and diverse patient sample from the Asia-Pacific region, with an age threshold of 50 years designated as a independent variable. The analysis also considered the mechanism and site of trauma as additional variables, while mortality and functional status were evaluated hierarchically to determine outcomes. The findings indicated that, within the Asia-Pacific cohort, trauma outcomes—including mortality and functional status—did not exhibit significant gender differences; rather, they were more closely associated with the severity of the anatomical site affected. This observation challenges the prevailing theoretical perspective that women typically experience better trauma outcomes. Such findings are crucial for informing the future development of more tailored trauma treatment and rehabilitation programs.

However, it is essential to acknowledge that infection plays a significant role in the outcomes of trauma [2]. A compromised integumentary barrier is unable to inhibit the infiltration of pathogenic bacteria, thereby rendering antimicrobials a crucial component in the management of traumatic injuries. Nevertheless, bacteria exhibit varying growth states at different temperatures, and despite the various interventions implemented to mitigate their proliferation, none can entirely negate the influence of temperature on bacterial colony growth [3].

While Bin Kunji Mohamad MI et al. examined the influence of various factors on trauma outcomes in their analysis, it is noteworthy that they did not account for seasonal variations or ambient temperature among the sample sources. We hope that this letter will positively influence the authors to consider a more nuanced analysis of the sample data, specifically by categorizing and quantifying samples based on the corresponding season and temperature conditions.

## Data Availability

No datasets were generated or analysed during the current study.
